# Time- and Dose-Dependent Effects of 17 Beta-Estradiol on Short-Term, Real-Time Proliferation and Gene Expression in Porcine Granulosa Cells

**DOI:** 10.1155/2017/9738640

**Published:** 2017-02-27

**Authors:** Sylwia Ciesiółka, Joanna Budna, Karol Jopek, Artur Bryja, Wiesława Kranc, Sylwia Borys, Michal Jeseta, Adrian Chachuła, Agnieszka Ziółkowska, Paweł Antosik, Dorota Bukowska, Klaus P. Brüssow, Małgorzata Bruska, Michał Nowicki, Maciej Zabel, Bartosz Kempisty

**Affiliations:** ^1^Department of Histology and Embryology, Poznan University of Medical Sciences, 6 Święcickiego St., 60-781 Poznań, Poland; ^2^Department of Anatomy, Poznan University of Medical Sciences, 6 Święcickiego St., 60-781 Poznań, Poland; ^3^Department of Obstetrics and Gynecology, University Hospital and Masaryk University, Obilni trh 11, 602 00 Brno, Czech Republic; ^4^Department of Anatomy and Histology, Faculty of Medicine and Health Sciences, University of Zielona Gora, Ul. Zyty 28, 65-046 Zielona Gora, Poland; ^5^Institute of Veterinary and Animal Science, Poznan University of Life Sciences, 52 Wojska Polskiego St., 60-628 Poznań, Poland

## Abstract

The key mechanisms responsible for achievement of full reproductive and developmental capability in mammals are the differentiation and transformation of granulosa cells (GCs) during folliculogenesis, oogenesis, and oocyte maturation. Although the role of 17 beta-estradiol (E2) in ovarian activity is widely known, its effect on proliferative capacity, gap junction connection (GJC) formation, and GCs-luteal cells transformation requires further research. Therefore, the goal of this study was to assess the real-time proliferative activity of porcine GCs in vitro in relation to connexin (Cx), luteinizing hormone receptor (LHR), follicle stimulating hormone receptor (FSHR), and aromatase (CYP19A1) expression during short-term (168 h) primary culture. The cultured GCs were exposed to acute (at 96 h of culture) and/or prolonged (between 0 and 168 h of culture) administration of 1.8 and 3.6 *μ*M E2. The relative abundance of Cx36, Cx37, Cx40, Cx43, LHR, FSHR, and CYP19A1 mRNA was measured. We conclude that the proliferation capability of GCs in vitro is substantially associated with expression of Cxs, LHR, FSHR, and CYP19A1. Furthermore, the GC-luteal cell transformation in vitro may be significantly accompanied by the proliferative activity of GCs in pigs.

## 1. Introduction

Proper mammalian oocyte development depends on bidirectional communication via gap junction connections (GJC) occurring between the female gamete and surrounding somatic cumulus cells (CCs) [1]. It is well known that connexins (Cxs) are the main markers of cumulus cell-oocyte crosstalk and therefore play a crucial role in the bidirectional transport of small molecules between these cells [2]. Moreover, it was recently reported that connexin 43 (Cx43) plays a critical role in this communication and may be recognized as the oocyte's maturation marker [3]. This paracrine association determines proper oocyte maturation and achievement of the MII stage. It has been shown that GJC are responsible for proper oocyte growth; however, it is still not entirely known how these communications influence granulosa-cumulus cell differentiation and proliferation in vitro.

Until now, there are few reports that indicate the ability of granulosa cells (GCs) to proliferate in vitro. Recently, we have shown that cumulus cells proliferate in vitro in cumulus-enclosed and cumulus-separated culture models [4]. Moreover, the subcellular distribution of maturation markers, Cdk4 and Cx43, differs significantly in follicles of various size and culture conditions. Short-term, in vitro proliferation of CCs significantly influenced Cdk4 and Cx43 protein expression, as well as their nuclear and cytoplasmic localization. However, the molecular factor that determines in vitro GC proliferation capability, as well as the GC-luteal cell transformation as related to the subcellular distribution of marker proteins, still needs further investigation.

It is well known that the mammalian ovary plays two important functions: the production of female gametes and synthesis of ovarian steroid hormones such as estrogen and progesterone. Both of these hormones are involved in hormonal feedback pathways that regulate follicular cell growth and differentiation. Moreover, it is a well-recognized theory that the ability of the ovary to synthetize 17 beta-estradiol (E2) is the main indicator of proper ovarian follicle function and activity. The androgens produced in theca cell layers via conversion are used in the synthesis of estrogen in granulosa cells. Moreover, GCs produce estrogen, and exposure to exogenous estrogen, due to administration of E2, might trigger negative feedback of estrogen in granulosa cells [5].

Since it was found that E2 is actively synthetized in GCs, it is hypothesized that E2 may play an important role in GC proliferation and GC-CC differentiation [6]. However, relation of Cxs expression with in vitro GCs proliferation in medium supplemented with E2 is still not fully recognized. Therefore, this study aimed to investigate porcine GC real-time proliferation after E2 treatment in relation to Cxs gene expression, addressing the role of GJC communication in real-time proliferation of E2 treated porcine GCs.

## 2. Material and Methods

### 2.1. Animals

A total of 40 pubertal crossbred Landrace gilts, bred on a local, commercial farm, were used for this study. They had a mean age of 155 days (range: 140–170 days) and a mean weight of 100 kg (95–120 kg). All animals were housed under identical conditions and fed the same forage (depending on age and reproductive status). The experiments were approved by the Local Ethics Committee.

### 2.2. Collection of Porcine Ovaries and In Vitro Cultivation of Porcine Granulosa Cells (GCs)

Ovaries and reproductive tracts were obtained at the time of slaughter and transported to the laboratory within 40 min at 38°C in 0.9% NaCl. To provide optimum conditions, the ovaries of each animal were placed in 5% fetal bovine serum (FBS; Sigma-Aldrich Co., St. Louis, MO, USA) in PBS [7, 8]. Granulosa cells were collected by aspirating the follicular fluid from single follicles using a 20 G needle. Collected cells were washed twice by centrifugation at 200 ×g for 10 min at RT with culture medium. COCs were removed from the cell suspension by filtration through a 40 *μ*m cell strainer and discarded. Medium consisted of Dulbecco's Modified Eagle's Medium (DMEM/F12, Sigma-Aldrich, USA), 2% fetal calf serum FCS (PAA, Austria), 10 mg/mL ascorbic acid (Sigma-Aldrich, USA), 0.05 *μ*M dexamethasone (Sigma-Aldrich, USA), 200 mM L-glutamine (Invitrogen, USA), 10 mg/mL gentamicin (Invitrogen, USA), 10,000 units/mL penicillin, and 10,000 *μ*g/mL streptomycin (Invitrogen, USA). Cells were cultivated at 38.5°C under aerobic conditions (5% CO_2_). Once adherent cells were more than 80% confluent, they were detached with 0.05% trypsin-EDTA (Invitrogen, USA) for 4 min and counted using a Z2 counter or cell viability analyzer Vi-Cell XR 2.03 (both Beckman Coulter, USA).

### 2.3. In Vitro GC Cultivation Using a Real-Time Cellular Analyzer (RTCA)

The recovered GCs were transferred to an E-Plate 16 of a real-time cell analyzer (RTCA, Roche-Applied Science, GmbH, Penzberg, Germany) consisting of an RTCA Analyzer, an RTCA SP Station, and RTCA software. The GCs were then cultured in 200 *μ*L of standard porcine IVM culture medium that consisted of Dulbecco's Modified Eagle's Medium (DMEM/F12, Sigma-Aldrich, USA) as mentioned above. The GCs were cultured for 0–168 h at 38.5°C under 5% CO_2_. The cultivation medium was replaced every 44 h. In the first experiment, GCs were supplemented with 1.8 and 3.6 *μ*M E2 at the beginning of the experiment (0 h). Simultaneously, in the second experiment, GCs were supplemented with the same doses of E2 at 96 h, during the logarithmic increase of proliferation (log phase). After every step of cultivation, the GCs were treated with trypsin (0.25% trypsin in a balanced salt solution, Sigma-Aldrich, St. Louis, MO, USA), and the cell index (CI) and normalized cell index were used to evaluate the relative and quantitative changes in electrical cell impedance. The cell status was determined using RTCA software.

### 2.4. RT-qPCR Analysis of Cx36, Cx37, Cx40, Cx43, LHR, FSHR, and CYP19A1 mRNA Expression in GCs

Total RNA was isolated from GCs before (at 0 h) and after (24, 48, 72, 96, 120, 144, and 168 h) IVC using an RNeasy mini column from Qiagen GmbH (Hilden, Germany). The RNA samples were resuspended in 20 *μ*L RNase-free water and stored in liquid nitrogen. RNA samples were then treated with DNase I and reverse-transcribed (RT) into cDNA. RT-qPCR was conducted using a LightCycler RT-qPCR detection system (Roche Diagnostics GmbH, Mannheim, Germany) with SYBR® Green I as a detection dye, and target cDNA was quantified using the relative quantification method. The relative abundance of Cx36, Cx37, Cx40, Cx43, LHR, FSHR, and CYP19A1 transcripts in each sample was standardized to the internal standard glyceraldehyde-3-phosphate dehydrogenase (GAPDH). For amplification, 2 *μ*L cDNA solution was added to 18 *μ*L of QuantiTect® SYBR Green PCR (Master Mix Qiagen GmbH, Hilden, Germany) and primers (Table 1). One RNA sample of each preparation was processed without the RT-reaction to provide a negative control for subsequent PCR.

To quantify specific genes expressed in the GCs, the expression levels of specific oocyte mRNAs in each sample were calculated relative to beta-actin (ACTB) and porphobilinogen deaminase (PBGD). To ensure the integrity of these results, the additional housekeeping gene 18S rRNA was used as an internal standard to demonstrate that ACTB and PBGD mRNAs were not differentially regulated in the groups of GCs. The expression of 18S rRNA has been identified as an appropriate housekeeping gene for use in quantitative PCR studies. Expression of ACTB, PBGD, and 18S rRNA mRNA was measured in cDNA samples from isolated GCs.

### 2.5. Statistical Analysis

A one-way ANOVA, followed by Tukey's post hoc test, was used to compare the results of the real-time quantification of the proliferation index. The experiments were carried out in at least two replicates. The results quantifying the cell proliferation index were obtained using an RTCA system. The differences were considered to be significant at ^*∗*^*P* < 0.05, ^*∗∗*^*P* < 0.01, and ^*∗∗∗*^*P* < 0.001 for the RT-qPCR and RTCA analyses, and they were evaluated by comparing the results obtained in five replicates of the same recovered granulosa cells. Statistical calculations were applied to the highest normalized proliferation index at each time point for each investigated group for comparative purposes. All statistical analyses were performed using the software program GraphPad Prism version 4.0 (GraphPad Software, San Diego, CA).

## 3. Results

Using an RT-qPCR assay, we analyzed the relative abundance of Cx36, Cx37, Cx40, Cx43, LHR, FSHR, and CYP19 mRNA after each period of GC real-time proliferation in vitro. Two housekeeping genes, ACTB and PBGD (data not shown), were used to normalize sample-to-sample RT-qPCR assays. The aim was to check whether mRNA expression profile changed among the groups. Additionally, two housekeeping genes were applied to check the total RNA isolation efficiency.

**Figure 1:**
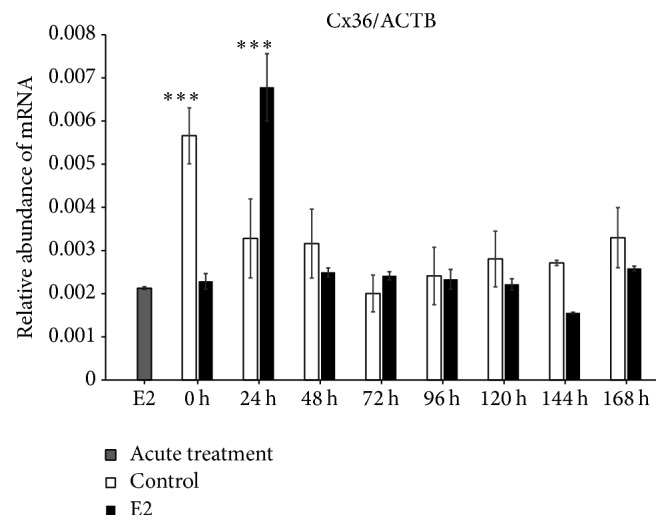
Relative abundance of Cx36 transcript in porcine follicular granulosa cells analyzed during 168 h of IVC. Porcine follicular granulosa cells were isolated from pubertal gilts and immediately used to isolate RNA, which was reverse-transcribed into cDNA. The relative abundance of Cx36 mRNA was evaluated by RT-qPCR analysis before and after each 24 h of IVC. The results are presented as the mean ± SEM with the level of significance shown as ^*∗∗∗*^*P* < 0.001.

**Figure 2:**
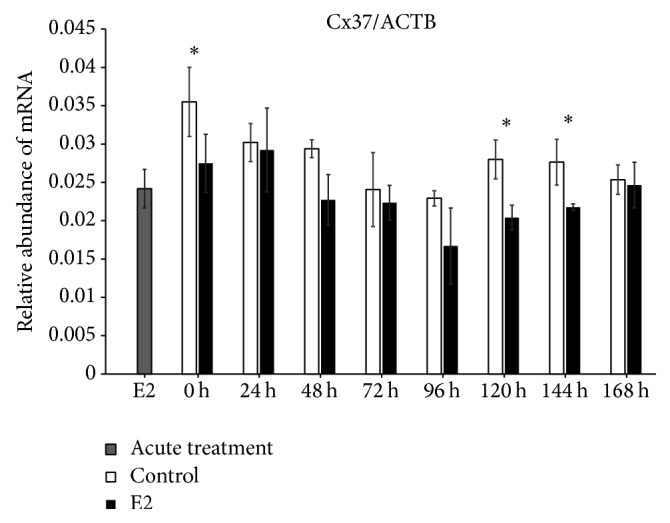
Relative abundance of Cx37 transcript in porcine follicular granulosa cells analyzed during 168 h of IVC. Porcine follicular granulosa cells were isolated from pubertal gilts and immediately used to isolate RNA, which was reverse-transcribed into cDNA. The relative abundance of Cx37 mRNA was evaluated by RT-qPCR analysis before and after each 24 h of IVC. The results are presented as the mean ± SEM with the level of significance shown as ^*∗*^*P* < 0.05.

**Figure 3:**
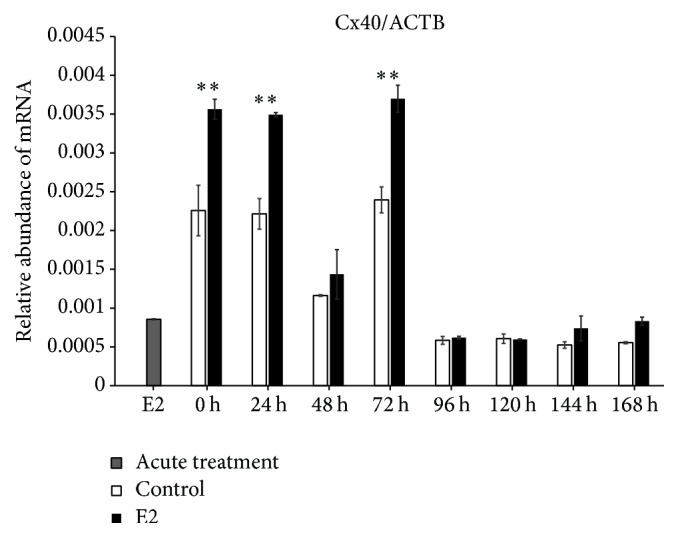
Relative abundance of Cx40 transcript in porcine follicular granulosa cells analyzed during 168 h of IVC. Porcine follicular granulosa cells were isolated from pubertal gilts and immediately used to isolate RNA, which was reverse-transcribed into cDNA. The relative abundance of Cx40 mRNA was evaluated by RT-qPCR analysis before and after each 24 h of IVC. The results are presented as the mean ± SEM with the level of significance shown as ^*∗∗*^*P* < 0.01.

**Figure 4:**
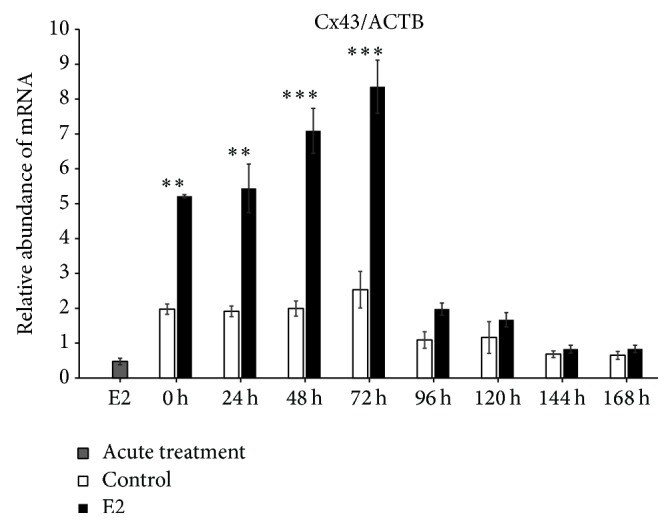
Relative abundance of Cx43 transcript in porcine follicular granulosa cells analyzed during 168 h of IVC. Porcine follicular granulosa cells were isolated from pubertal gilts and immediately used to isolate RNA, which was reverse-transcribed into cDNA. The relative abundance of Cx43 mRNA was evaluated by RT-qPCR analysis before and after each 24 h of IVC. The results are presented as the mean ± SEM with the level of significance shown as ^*∗∗*^*P* < 0.01 and ^*∗∗∗*^*P* < 0.001.

**Figure 5:**
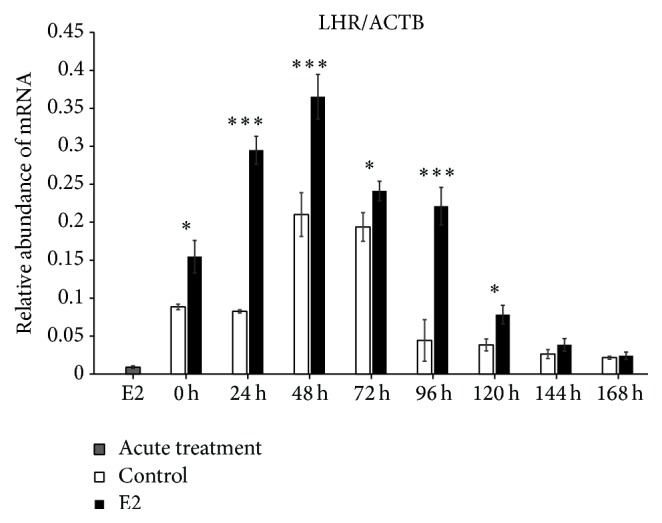
Relative abundance of LHR transcript in porcine follicular granulosa cells analyzed during 168 h of IVC. Porcine follicular granulosa cells were isolated from pubertal gilts and immediately used to isolate RNA, which was reverse-transcribed into cDNA. The relative abundance of LHR mRNA was evaluated by RT-qPCR analysis before and after each 24 h of IVC. The results are presented as the mean ± SEM with the level of significance shown as ^*∗*^*P* < 0.05 and ^*∗∗∗*^*P* < 0.001.

**Figure 6:**
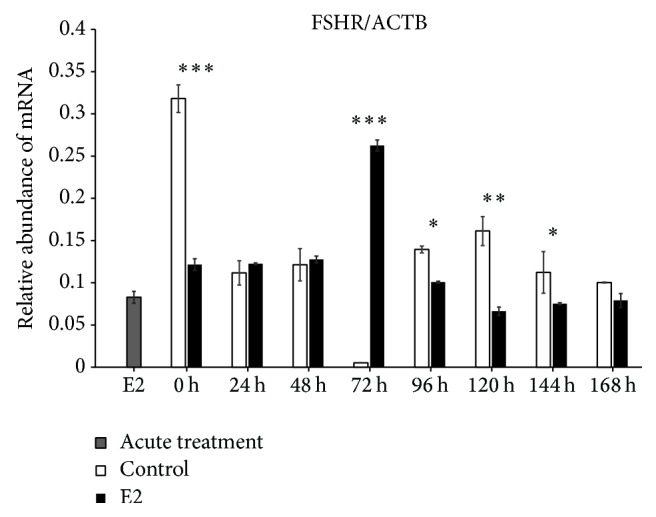
Relative abundance of FSHR transcript in porcine follicular granulosa cells analyzed during 168 h of IVC. Porcine follicular granulosa cells were isolated from pubertal gilts and immediately used to isolate RNA, which was reverse-transcribed into cDNA. The relative abundance of FSHR mRNA was evaluated by RT-qPCR analysis before and after each 24 h of IVC. The results are presented as the mean ± SEM with the level of significance shown as ^*∗*^*P* < 0.05, ^*∗∗*^*P* < 0.01, and ^*∗∗∗*^*P* < 0.001.

**Figure 7:**
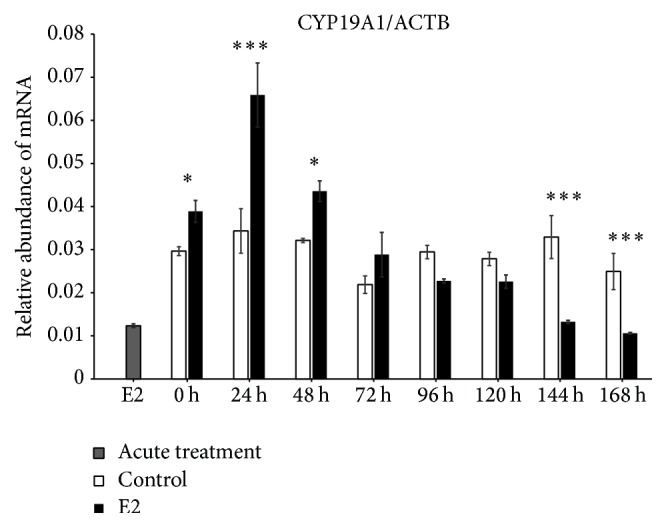
Relative abundance of CYP19A1 transcript in porcine follicular granulosa cells analyzed during 168 h of IVC. Porcine follicular granulosa cells were isolated from pubertal gilts and immediately used to isolate RNA, which was reverse-transcribed into cDNA. The relative abundance of CYP19A1 mRNA was evaluated by RT-qPCR analysis before and after each 24 h of IVC. The results are presented as the mean ± SEM with the level of significance shown as ^*∗*^*P* < 0.05 and ^*∗∗∗*^*P* < 0.001.

We found increased expression of Cx36 mRNA in control (untreated cells) at 0 h of IVC (*P* < 0.001), whereas, after 24 h of IVC, a higher level of this transcript was observed in the prolonged E2 treated group (*P* < 0.001). Between 48 and 168 h, we did not determine differences between the groups. The acute E2 treatment displayed a similar decreased effect on Cx36 mRNA expression (Figure 1). The Cx37 transcript revealed increased expression at 0, 120, and 144 h in the control group as compared to the prolonged E2 treated group (*P* < 0.05) (Figure 2). The Cx40 mRNA had elevated expression in the prolonged E2 treated group at 0, 24, and 72 h as compared to untreated controls (*P* < 0.01) (Figure 3). Similarly, Cx43 displayed higher expression at 0 and 24 h in the E2 treated group (*P* < 0.01) and at 48 and 72 h (*P* < 0.001) compared to untreated controls (Figure 4). The LHR mRNA was increased at 0, 24, 48, 72, 96, and 120 h in the prolonged E2 treated group as compared to controls (*P* < 0.05 and *P* < 0.001, resp.) (Figure 5). Conversely, the relative abundance of FSHR mRNA was increased at 72 h in the E2 treated group (*P* < 0.001). The FSHR transcript was more highly expressed in control at 0, 96, 120, and 144 h as compared to the E2 treated group (*P* < 0.001, *P* < 0.01, and *P* < 0.05, resp.) (Figure 6). Similar to LHR, the abundance of CYP19A1 mRNA was increased at 0, 24, and 48 h in the E2 treated group as compared to controls (*P* < 0.05, *P* < 0.001), but increased CYP19A1 transcript levels were observed at 144 and 168 h in controls (Figure 7).

**Figure 8:**
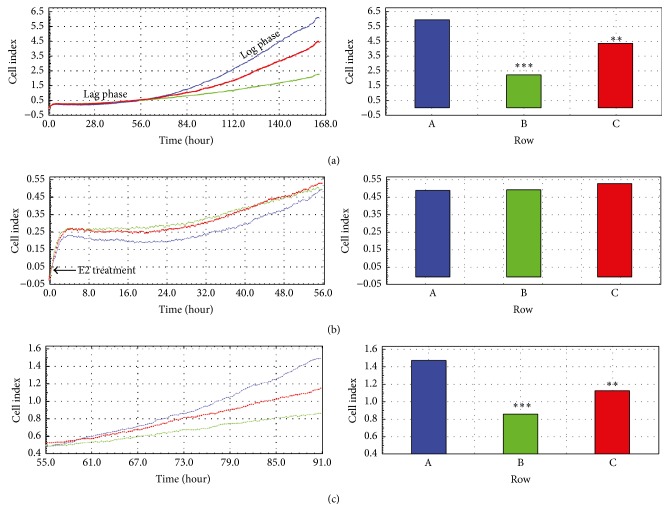
Cell proliferation index (CI) of porcine follicular granulosa cells cultivated for 168 h after acute and prolonged E2 treatment. Porcine follicular granulosa cells were recovered from pubertal gilts and treated with collagenase for 10 min at 38.5°C. The cells were immediately transferred into an E-Plate 48 of a real-time cell analyzer (RTCA, Roche-Applied Science, GmbH, Penzberg, Germany). The experiment consisted of eight replicates involving the cultivation of the same population of collected cells. In the first experiment, the porcine GCs were cultured with 1.8 and 3.6 *μ*M E2 at the beginning of an experiment (0 h), which was divided into three time periods: 0–168 h (Figure 8(a)), 0–56 h (Figure 8(b)), and 56–90 h (Figure 8(c)). The differences were considered to be significant at the level of ^*∗∗*^*P* < 0.01 and ^*∗∗∗*^*P* < 0.001. A, B, and C represent triplicates (three samples of the same population of collected cells).

Using the RTCA system, the proliferation index (PI) and effects of acute and prolonged E2 treatment were assessed. In the first experiment, the porcine GCs were cultured with 1.8 and 3.6 *μ*M E2 at the beginning of an experiment (0 h), which was divided into three time periods: 0–168 h (Figure 8(a)), 0–56 h (Figure 8(b)), and 56–90 h (Figure 8(c)). When analyzing the complete culture period, we found a significant increase in PI in the control group as compared to 1.8 and 3.6 *μ*M E2 treated groups (*P* < 0.001 and *P* < 0.01, resp.) (Figure 8(a)). Between 0 and 56 h of IVC, no differences in PI value were observed (Figure 1). However, during the last cultivation period, higher PI in the control group was determined for both doses of 1.8 and 3.6 *μ*M E2 (*P* < 0.001 and *P* < 0.01, resp.) (Figure 8(c)).

**Figure 9:**
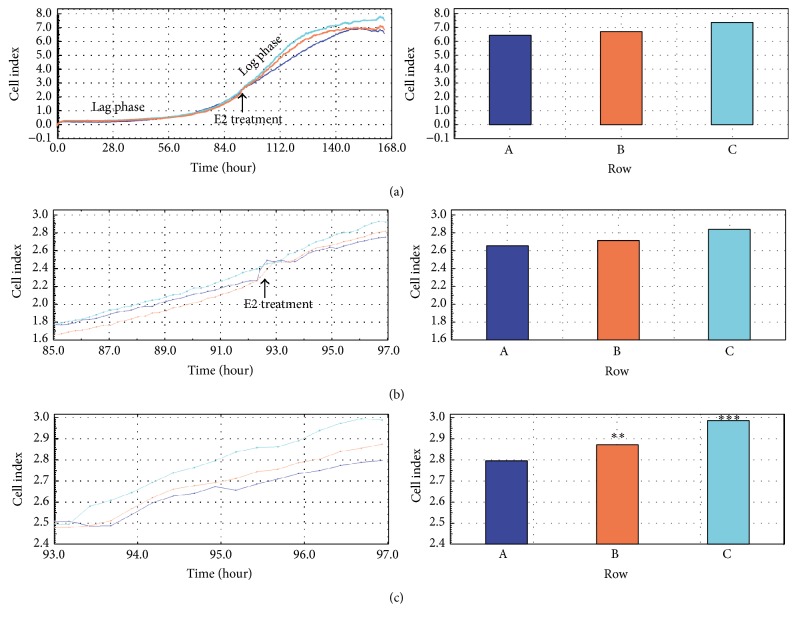
Cell proliferation index (CI) of porcine follicular granulosa cells cultivated for 168 h with acute E2 treatment during the logarithmic increase of proliferation. Porcine follicular granulosa cells were recovered from pubertal gilts and treated with collagenase for 10 min at 38.5°C. The cells were immediately transferred into an E-Plate 48 of real-time cell analyzer (RTCA, Roche-Applied Science, GmbH, Penzberg, Germany). The experiment consisted of eight replicates involving the cultivation of the same population of collected cells. Similarly, the PI was evaluated at three different time periods, that is, 0–168 h (Figure 9(a)), 85–97 h (Figure 9(b)), and 93–97 h (Figure 9(c)). The porcine GCs were treated with 1.8 and 3.6 *μ*M E2 at 96 h of culture (Figure 9(b)). The differences were considered to be significant at the level of ^*∗∗*^*P* < 0.01 and ^*∗∗∗*^*P* < 0.001. A, B, and C represent triplicates (three samples of the same population of collected cells).

In the second experiment, the effect of acute E2 treatment on GCs during the logarithmic increase of proliferation was assessed. Similarly, the PI was evaluated at three different time periods. When analyzing complete culture periods 0–168 h, we did not observe differences in PI between the control and treatment groups (Figure 9(a)). The GCs were treated with 1.8 and 3.6 *μ*M E2 at 96 h of culture (Figure 9(b)). Subsequently, we observed a significant increase of the PI after 1.8 and 3.6 *μ*M E2 treatment as compared to the control (*P* < 0.01 and *P* < 0.001, resp.) (Figure 9(c)).

## 4. Discussion

Mammalian cumulus-oocyte complex (COC) maturation, both in vivo and in vitro, belongs to compound processes orchestrated by substantial morphological and molecular changes occurring within denuded oocytes and surrounding somatic cumulus-granulosa cells [9]. These modifications involve (1) accumulation of mRNAs and proteins within the oocyte's cytoplasm (cytoplasmic maturation), (2) chromosomal reorganization (nuclear maturation), (3) cumulus cell (CC) expansion, and (4) activation of CC-oocyte bidirectional contact via gap junction connections (GJC). It has been observed in several mammalian species that proper crosstalk is necessary for progressive oocyte growth and CC proliferation and differentiation [10, 11]. Moreover, the lack of cumulus oophorus layers and/or an expanded cumulus after maturation are main causes of disturbance during fertilization and zygote formation [12]. The GJC peptide structure is composed of several connexins (Cxs), which are responsible for small substances shuttling between CCs and oocytes. Our previous results indicated the expression and possible function of Cxs in porcine GCs during short-term, real-time primary culture [2]. Porcine follicular GCs were used as the study model, where the effect of E2 on Cxs (Cx36, Cx37, Cx40, and Cx43) expression pattern during GCs real-time, short-term in vitro cultivation (IVC) was assessed. During maturation of mammalian COCs, both in vivo and in vitro, cumulus cells that tightly surround the oocyte undergo significant morphological and biochemical modifications, a process also known as cumulus expansion. However, it still remains unclear whether this unique process is accompanied by CC proliferation. Our recent studies clearly demonstrated that porcine cumulus-enclosed oocytes, separated CCs, or GCs successfully proliferate in vitro during short-term, real-time primary culture [4, 13]. However, the acute and/or prolonged effect of E2 administration on the intensity of porcine GC proliferation in vitro has yet to be investigated. We found that prolonged treatment of E2, used in doses of 1.8 and 3.6 *μ*M, leads to decreased GC proliferation intensity, when the cells enter the log phase of proliferation between 72 and 96 h of IVC. However, acute treatment of E2 results in an increased PI after both doses of the hormone. It remains unclear whether various time periods of E2 administration display differential effects on GC proliferation. We suggested that the temporal alterations in intensity of porcine GCs' in vitro proliferation may be associated with specialization and increased specificity or cell viability after 72 h of IVC. It has been well recognized that GCs undergo substantial transformation into luteal cells after 48–72 h of in vitro culture. Therefore, it may be postulated that the differential effect of acute E2 treatment is orchestrated by the specific response of GCs to hormonal stimulation. Although the steroid hormones profile was not assessed in the study, we suggest that the prolonged effect of E2 treatment may act via different mechanisms during GC-luteal cell transformation and/or significantly alter this process in vitro.

Minimal data exists indicating CC expansion is associated with significant changes in the biochemical prolife of target gene expression. In the current study, we assessed the expression profile of Cxs, which function as CC-oocyte communication markers, and the activity of GJC during short-term, real-time cell proliferation. We found that the profile of Cxs expression during 168 h of IVC was substantially changed. Furthermore, acute and/or prolonged effect of E2 supplementation on cell proliferation was also observed. It is widely known that E2 is crucial for proper functions of reproductive processes in mammals, especially in folliculogenesis and oogenesis regulation. Moreover, estrogen, produced by mature ovarian follicle, is a noteworthy factor in the proliferative phase of the endometrium. However, it is not fully recognized if E2 influences GJC activity via upregulation of Cx expression. The results from the current study clearly demonstrated that E2 may modulate Cx expression after acute and/or prolonged treatment, and it may be the potential factor associated with paracrine activity of both oocytes and CCs during the induction of gamete-somatic cell “crosstalk.”

The role of estrogen and progesterone hormones on Cx expression in various types of ovarian tissue was recently investigated in several mammalian species [14, 15]. However, the effect of E2 on the Cx expression profile in GCs and CCs is unknown. The role of Cxs in regulation of growth, oocyte development, and differentiation of surrounding CCs via the induction activity of GJC is widely recognized [16]. It is also well known that this process is regulated by factors, that is, hormones, which act via paracrine secretion. However, the mechanisms that regulate GJC activity and/or communication pathways between an oocyte and CCs still need exploration.

Recently, Saadeldin et al. investigated the interaction between porcine denuded oocytes and GCs in juxtacrine and paracrine bidirectional interaction models in relation to Cx43 expression [17]. They used a transwell 0.4 *μ*m polyester membrane insert system to permit an oocyte-granulosa cell paracrine interaction in a coculture model. The higher rate of blastocyst parthenogenetic formation was observed in a direct communication model as compared to paracrine model. Interestingly, the changes in steroid profiles such as P4 and E2, and mRNA expression of enzymes crucial for steroidogenesis after 22 and 44 h of juxtacrine and paracrine communication models, were also determined. The expression of Cx43 was significantly higher in a GC direct contact model than in an indirect coculture system. These results confirm previous observation that a direct contact model for in vitro culture of GCs revealed better growth conditions for oocytes and differentiation of GCs.

Similarly, our results indicated an association between the PI during short-term, porcine GCs in vitro culture and the expression profile of selected Cxs in relation to the stimulatory effect of E2 administration. In our recent study, we investigated the expression of selected connexins such as Cx36, Cx37, Cx40, and Cx43 mRNA by RT-qPCR as well as distribution of Cx30, Cx31, Cx37, Cx43, and Cx45 proteins in porcine GCs during short-term (168 h), real-time cell proliferation in vitro [2]. We observed increased expression of Cx36, Cx37, and Cx43 mRNAs in GCs at recovery as compared to all analyzed time periods of IVC (24, 48, 72, 96, 120, 144, and 168 h; *P* < 0.001). Moreover, the expression patterns of Cx31, Cx37, and Cx45 proteins were increased before (0 h) as compared to after 168 h of IVC. Similarly, the PI value was most increased between 72 and 96 h of IVC. Taking into account both these results, we suggest that the decreased expression of Cxs and/or GJC breakdown during IVC may be significantly associated with the differentiation of GCs into luteal cells in vitro.

Testosterone's effect on 17 beta-estradiol secretion as related to aromatase activity was previously researched using a GC rat model by Lee et al. [18]. The authors found that testosterone treatment (0.1 and 1 *μ*g/mL) increases aromatase activity and secretion of 17 beta-estradiol. Moreover, higher doses of testosterone downregulate Cx43 expression in rat GCs, which was reversed by supplementation with calcitriol (1,25-D3). Our study demonstrated that CYP19A1 mRNA was significantly increased after E2 treatment. In our current research, we did not assess testosterone concentration or the effect of its supplementation on CYP19A1 expression. However, the upregulatory effect of E2 administration on aromatase expression was observed. The results by Lee et al. are in agreement with our findings since positive regulation of aromatase expression was found. This may be an effect of phosphotyrosine stimulation, thereby increasing the concentration and availability of 1,25-D3, which is the potent factor of testosterone-induced 17 beta-estradiol production in GCs.

The function of GCs isolated from oocytes of young, middle-aged, and older infertile patients, as related to steroidogenesis, apoptosis, and gonadotropin activity during ovarian ovulation, was recently investigated by Wu et al. Similar to our study, GCs were collected form ovarian follicular fluid and the expression of FSHR, CYP19A1, HSD17B, LHR, CYP11A1, and PGR (progesterone receptor) was assessed. They found that FSHR, CYP19A1, and HSD17B expression was decreased in GCs isolated from aged patients, whereas LHR, CYP11A1, and PGR were stimulated in this group. Moreover, the in vitro proliferation of GCs was lower, and the rate of apoptosis was increased in aged patients. The premature luteinization was accompanied by an age-related decline of GC function in vitro, whereas addition of FSH to the culture prevented GC luteinization [19].

The results of the current study indicated increased expression of both gonadotropin receptors (LHR and FSHR) at 0–24 h in untreated control, whereas upregulation of these genes at 48–72 h after E2 administration was observed. It is suggested that E2 treatment reflects a stimulatory effect on GCs in vitro proliferation, the ability to reach log phase at 72 h, and expression of LHR. Moreover, LHR expression may be recognized as a GC-luteal cell transformation marker since the highest expression level was observed between 48 and 72 h. Additionally, the upregulation of FSHR at 72 h of IVC is also accompanied by a substantial increase of porcine GC proliferation in vitro. It is widely known in several mammalian species that primary culture of GCs and/or research based on GC-luteal cell transformation is a reliable and convenient model for ovarian folliculogenesis and may be recognized as a specific “fingerprint” of this process.

Baufeld and Vanselow investigated the morphological and physiological properties of bovine GCs in vitro in relation to luteinization marker expression and methylation status [20]. They found that CYP19A1 is the crucial enzyme in the pathway of estradiol production, and expression of this enzyme is associated with follicular size. Only the GCs isolated from larger size follicles were characterized by increased specificity and sensitivity to the ovarian action of LH. Moreover, they observed a significant association between 17 beta-estradiol synthesis and CYP19A1 expression; however, the proliferative activity of GCs, as measured by Proliferating Cell Nuclear Antigen (PCNA) expression, was reversible and correlated with the expression of steroidogenesis markers and gonadotropin receptors. The decreased expression of luteinization markers such as Prostaglandin-Endoperoxide Synthase 2 (PTGS2), Pentraxin 3 (PTX3), Regulator of G-Protein Signaling 2 (RGS2), and Vanin 2 (VNN2) was also determined. Contrary to those results, our findings demonstrated a positive correlation between E2 administration and proliferative capability of porcine GCs cultured in vitro. However, the stimulatory effect of E2 on CYP19A1 was similarly observed. What is more, in our recent study, we have presented relation of GCs luteinization with expression of LHR, FSHR, and CYP19A1 mRNA and distribution of proteins encoded by this genes in in vitro culture [21]. LHR and CYP19A1 were increased at 24 and 48 h of IVC, whereas FSHR obtained the highest level at 0 h. Similarly, an increased PI was noted soon after 24 h of IVC. It was assumed that the physiological state of GCs proliferation is not associated with expression of luteinization markers. Comparing these results with the current study, we postulate that only E2-stimulated GCs are correlated with an increase of PI, which may be accompanied by a substantial upregulation of GC-luteal cell transformation in vitro, even if some GCs can be negatively regulated by extensive estrogen concentration due to exogenous E2 administration. Moreover, connexins expression, which is a marker of GJC activity, may be modulated by E2 supplementation.

## Figures and Tables

**Table 1:** Oligonucleotide sequences used for RT-qPCR analysis.

Gene	Gene accession number	Primer sequence (5′-3′)	Product size (bp)
CYP19	NM_214429.1	AAAGACGCAGGATTTTCACA TCTTTTGTCAGTTCACCACGT	97
FSHR	NM_214386.2	GAATTGAAAAGGCCAACAAC CTTTCAAAACTTAGTCCCACG	214
LHR	NM_214449.1	AAGCACAGCAAGGAGACCAA AAGAGGACAGTCACGTTTCC	231
Cx36	XM_001924757.4	AGCAGCACTCCACTATGATCGG TGTTGCACACAAACATGGTCT	123
Cx37	NM_001244224.1	GTGTGTCCGCACCGCCACCGATGA ACAGGACCGTCAGCCAGA	155
Cx40	XM_003130802.2	ATCGCTTTCACCTGCAAGTCC CGCCATCCTCAGCAGAACCAT	220
Cx43	NM_001244212.1	CAGCACTTTTCTTTCATTAGGGGC CTAAGGACTCCAGTCACC	194
ACTB	XM_003124280.3	CCCTTGCCGCTCCGCCTTC GCAGCAATATCGGTCATCCAT	69
PBGD	NM_001097412.1	GAGAGTGCCCCTATGATGCT ATGATGGCACTGAACTCCT	214
